# Genetic characterization of commensal *Escherichia coli* isolated from laboratory rodents

**DOI:** 10.1186/s40064-016-2745-9

**Published:** 2016-07-11

**Authors:** Shih Keng Loong, Nur Hidayana Mahfodz, Nurul Asma Anati Che Mat Seri, Haryanti Azura Mohamad Wali, Syahar Amir Abd Gani, Pooi-Fong Wong, Sazaly AbuBakar

**Affiliations:** Tropical Infectious Diseases Research and Education Centre, Faculty of Medicine, University of Malaya, 50603 Kuala Lumpur, Malaysia; Animal Experimental Unit, Faculty of Medicine, University of Malaya, 50603 Kuala Lumpur, Malaysia; Department of Pharmacology, Faculty of Medicine, University of Malaya, 50603 Kuala Lumpur, Malaysia; Department of Medical Microbiology, Faculty of Medicine, University of Malaya, 50603 Kuala Lumpur, Malaysia

**Keywords:** *Escherichia coli*, Commensal, Malaysia, Rodent

## Abstract

**Background:**

*Escherichia coli*, a commensal in the intestines of vertebrates, is capable of colonizing many different hosts and the environment. Commensal *E. coli* strains are believed to be the precursor of pathogenic strains by means of acquisition of antimicrobial resistant and virulence genes. Laboratory rodents are inherently susceptible to numerous known infectious agents, which could transfer virulence determinants to commensal *E. coli*. Hence, in this study, the genetic structure of commensal *E. coli* found in laboratory rodents and their antimicrobial resistance profiles were investigated.

**Results:**

*E. coli* strains belonging to phylogroup A were the predominant strain obtained from the animals used in the study. Four novel sequence types (ST746, ST747, ST748 and ST749) were discovered using the multi locus sequence typing, together with one common ST357 in the gastrointestinal tract, liver and, the trachea and lung. Serotyping demonstrated that these commensal *E. coli* strains were non-Shiga toxin-producers. Phenotypic and genotypic analyses of extended spectrum beta lactamases were also negative.

**Conclusions:**

These findings implied that the *E. coli* strains recovered from the laboratory rodents were truly commensal in nature. Further study is required to investigate the possible influence of gender on the susceptibility of hosts to *E. coli* colonization in laboratory rodents.

**Electronic supplementary material:**

The online version of this article (doi:10.1186/s40064-016-2745-9) contains supplementary material, which is available to authorized users.

## Background

*Escherichia coli*, a non-sporulating, Gram-negative, rod-shaped aerobe, is a commensal in the intestines of vertebrates (Duriez et al. 2001; Escobar-Páramo et al. 2006; Jaureguy et al. 2008; Tenaillon et al. 2010; Schmidt et al. 2015), present in various habitats ranging from deserts to the arctic (Escobar-Páramo et al. 2006). This bacteria species also thrives in water environments, with half of the *E. coli* population estimated to reside in these secondary habitats (Tenaillon et al. 2010), facilitating anthropogenic transmission from species to species through water and food ingestion (Escobar-Páramo et al. 2006). These characteristics suggest that *E. coli* is capable of colonizing and spreading in different environments (Escobar-Páramo et al. 2006). Pathogenic strains of *E. coli* can cause serious infections including, intestinal and extraintestinal diseases in humans (Tenaillon et al. 2010). Previous studies have suggested that commensal strains of *E. coli* may be the precursor to the pathogenic *E*. *coli* strains following acquisition of virulence genes (Duriez et al. 2001), potentially implicating commensal *E. coli* as the natural reservoir of pathogenic strains (Schmidt et al. 2015). Enhancement of pathogenicity through random point mutations or acquisition of chromosomal virulence operons were proposed to be methods by which commensal *E*. *coli* can become pathogenic (Duriez et al. 2001), and this was possible with *E. coli* having a clonal structure (Tenaillon et al. 2010; Smati et al. 2015) coupled with a high level of genome plasticity.

Phylogenetic analyses have greatly aided our understanding of the genetic structure within the *E. coli* species by the assignment of at least seven phylogroups belonging to *E. coli* sensu stricto (A, B1, B2, C, D, E and F) (Clermont et al. 2013) and the multi locus sequence typing (Jaureguy et al. 2008). Serotyping via molecular typing of the O-antigens could also assist in the study of the genetic structure of *E. coli* (Sánchez et al. 2015). These methods are powerful tools for determining the phylogenetic relationships among the different *E. coli* lineages and could possibly provide greater understanding of the factors that shape the genetic structure of *E. coli* (Jaureguy et al. 2008; Clermont et al. 2013). However, the vast majority of research focus have been biased towards human *E. coli* strains. Laboratory rodents have been pivotal as a model for the study of the complex physiological system of mammals. They have contributed immensely to our understanding of the various human diseases, enhancing our knowledge to find effective treatments for these diseases (Jacoby and Lindsey 1998). However, these rodents are also inherently susceptible to numerous known bacterial, fungal and viral diseases (Jacoby and Lindsey 1998). Some of these infectious agents cause subclinical and unnoticeable infections that could nevertheless, adversely affect biological experimental data (Jacoby and Lindsey 1998; Loong et al. 2016).

Animal husbandry practices can potentially alter the composition of laboratory rodent microbiota, affecting experimental outcomes by introducing variables into the animals (Ma et al. 2012). The gastrointestinal microbiota are associated with the regulation of innate immunity and pathogen recognition receptors (Lee et al. 2011). Accordingly, the microbiota has been shown to modulate pro- and anti-inflammatory immune responses in mice models of multiple sclerosis (Lee et al. 2011) and type-1 diabetes (Burrows et al. 2015), suggesting that perturbation of the fragile equilibrium within the gastrointestinal tract impacts the pathologies of mice disease models. Upsetting of the normal microbiota by dysbiosis, abolishes colonization resistance, disrupts homeostasis and increases the host’s susceptibility to diseases (Ma et al. 2012). The successful mice intestine colonization by *Campylobacter jejuni* after antibiotics elimination of the gastrointestinal microbiota (O’Laughlin et al. 2015) demonstrated the effect of the abolishment of colonization resistance. A recent survey in 21 different German animal facilities found that shed skin or dust particles carried by animals, care takers or scientists influenced the gastrointestinal microbiota of experimental mice (Rausch et al. 2016), potentially affecting experimental outcomes. Fluctuations in the laboratory rodent microbiota as a result of external stimuli has to be reduced or eliminated to ensure consistency and reproducibility of experimental results. Therefore, surveillance of commensal *E. coli* populations in laboratory rodents could provide an indication of possible alteration of the normal gastrointestinal microbiota.

As *E. coli* is widely used as an indicator of antimicrobial selection pressure (Grønvold et al. 2010) and newly introduced bacteria species may transfer antimicrobial resistant determinants to commensal species (Schmidt et al. 2015), assessment of the genetic characteristics of commensal *E. coli* would provide information on the virulence status in the animals housed in the animal experimental facility. The occurrence of horizontal gene transfer of beta-lactamases among *E. coli* (Jaureguy et al. 2008; Koga et al. 2015) and the reverse transfer of antimicrobial resistance from humans to animals (Grall et al. 2015) could transpire as a result of the close interaction between scientists (humans) and laboratory rodents (animals). Hence, determination of antimicrobial resistance at the genetic level is important for understanding and limit antimicrobial resistance. The aims of this study were to determine the genetic structure of *E. coli* strains from the organs of otherwise healthy laboratory rodents to establish a background for further studies on pathogenic strains and to analyze the antimicrobial resistance profiles of the isolated *E.coli* strains.

## Methods

### Isolation of *E. coli*

A total of 67 randomly selected healthy rodents (mice; n = 38 and rats; n = 29) aged between 6 and 12 weeks old, housed in the Animal Experimental Unit, Faculty of Medicine, University of Malaya during March 2014–April 2015, were assessed as part of the unit’s rodents health monitoring program (Table 1). The sex of the rodents were distributed quite proportionately between males (n = 32) and females (n = 35) (Table 1). Healthy mice were euthanized and serologically assayed for selected pathogens according to previously published protocols (Loong et al. 2016). The rats however, were anaesthetized with Ketamine (50 mg/kg) and Xylazine (5 mg/kg) prior to blood collection through cardiac puncture. The collected rat blood was tested by serology (SMART-SPOT Sentinel Panel Test, Rat Sera, Biotech Trading Partners, USA) for the following agents; Kilham rat virus, *Mycoplasma pulmonis*, pneumonia virus of mice, rat coronavirus, respiratory enteric orphan virus, rat parvovirus, Sendai virus and Theiler’s murine encephalomyelitis virus. Following that, the rats were euthanized by overdosing with the same drugs at three times the anaesthetic dosage. Rodents were housed in open cages and other detailed procedures were described in a previous study (Loong et al. 2016). Selected rodent organs (gastrointestinal tract, liver and, the trachea and lung) were harvested, homogenized and cultured onto MacConkey agar. Organs from every rodent were cultured onto individual agar plate. Cultured bacterial strains suspected to be *E. coli* were identified using Gram staining, microscopy and 16S rDNA sequencing (Misbah et al. 2005). One strain was selected from each suspected culture plates after 24 h incubation at 37 °C for this study.

**Table 1:** Breed of rodents assessed between March 2014 and April 2015

Breed	Male (n)	Female (n)
Sprague–Dawley rat	10	10
Wistar Kyoto rat	3	3
Spontaneously hypertensive rat	2	1
ICR mouse	14	15
BALB/c mouse	3	3
C57BL/6 mouse	0	3
Total	67	

### Multi locus sequence typing (MLST) and phylogroup classification

Commensal *E. coli* strains were subjected to MLST (referred to as the Pasteur scheme), where the internal portions of eight housekeeping genes (*dinB*, *icdA*, *pabB*, *polB*, *putP*, *trpA*, *trpB* and *uidA*) were sequenced and compared with the database at http://bigsdb.web.pasteur.fr/ecoli/ecoli.html (Jaureguy et al. 2008). A specific allele number was assigned to each distinct sequence within a locus and a specific sequence type (ST) was assigned to each distinct combination of alleles. A novel allele number was assigned to sequences that could not be found in the database, subsequently establishing a novel ST. For phylogroup classification, *E. coli* strains were designated into the phylogroups; A, B1, B2, C, D, E, or F, according to a previous study (Clermont et al. 2013). This quadruplex PCR method differentiates the *E. coli* phylogroups by nucleic acid amplification of the *arpA*, *chuA* and *yjaA* genes, and the DNA fragment, TspE4.C2.

### Identification of Shiga toxin-producing *E. coli* serotypes

*E. coli* serogroups O5, O15, O26, O45, O55, O76, O91, O103, O104, O111, O113, O118, O121, O123, O128, O145, O146, O157, O165, O172 and O177 are the O-antigen types of the most clinically significant Shiga toxin-producing *E. coli* serotypes. These 21 serotypes were identified using three serotype-specific multiplex PCR assays, and it can also detect the respective O-antigens even when they are not expressed by the *E. coli* strains (Sánchez et al. 2015).

### Extended spectrum beta lactamases (ESBL) phenotypic confirmatory method

All commensal *E. coli* strains were cultured onto Mueller–Hinton agar after adjustment to 0.5 McFarland standards. Antimicrobial discs containing cefotaxime (30 μg), cefotaxime/clavulanic acid (30/10 μg), ceftazidime (30 μg) and ceftazidime/clavulanic acid (30/10 μg) were placed onto the agar plate so that the distances between them are approximately twice the radius of the inhibition zones, and the plate was incubated overnight at 35 °C. ESBL activity was indicated when a ≥5 mm increase in the inhibition zone diameter around either cephalosporin disc in combination with clavulanic acid as compared to the inhibition zone diameter of the cephalosporin when tested alone (CLSI 2012). The *E. coli* strain ATCC 25922 and *Klebsiella pneumoniae* strain ATCC 700603 were used as controls as per CLSI recommendation.

### Detection of genes encoding for ESBL

Five multiplex and one singleplex PCRs were employed in this study to detect genes encoding important beta-lactamases; one CTX-M multiplex PCR including groups 1, 2, 8, 9 and 25 (Woodford et al. 2006); one TEM/SHV/OXA-1-like multiplex PCR (Dallenne et al. 2010); one plasmid-mediated AmpC gene multiplex PCR including ACC, FOX, MOX, DHA, CIT and EBC (Dallenne et al. 2010); one VEB/GES/PER multiplex PCR (Dallenne et al. 2010); one VIM/IMP/KPC multiplex PCR (Dallenne et al. 2010); and one OXA-48-like singleplex PCR (Dallenne et al. 2010). In addition, the New Delhi metallo-beta-lactamase, NDM-1 was detected using PCR as previously described (Peirano et al. 2011).

### Detection of *E. coli* from the animal housing environment, rodent feed and drinking water

Testing for the presence of *E. coli* in the animal housing environment was performed by taking swabs from the tabletop, tap, trolley and wash basin in the room housing the rodents. Sterile, irradiated rodent feed (#1320-Maintenance diet for rats and mice, Altromin International, Germany) was vortexed in tubes containing nuclease-free water. Following that, the swabs, the vortexed feed and the autoclaved drinking water were cultured onto MacConkey agar and incubated at 37 °C under aerobic condition for 24 h. One colony resembling the morphological appearance of *E. coli* was selected from each positive plate and the *E. coli* phylogroup was determined. Additionally, detection of genes encoding for ESBL was also performed.

## Results

In the study period of 14 months (March 2014–April 2015), we randomly selected and examined a total of 67 healthy rodents of various breeds, housed in the AAALAC-certified facility at the Animal Experimental Unit, University of Malaya. This was performed as part of the health monitoring program to ensure that the rodents are free of specific pathogens that could affect scientific experiments. A total of 13 commensal *E. coli* strains were recovered on MacConkey agars, from the gastrointestinal tract, liver and, the trachea and lung of laboratory rodents (Table 2). Accession numbers for the partial 16S rDNA sequences of the 13 commensal *E. coli* strains include, LT009415–LT009427. All 13 commensal *E. coli* strains in this study were recovered from female rodents. These 13 strains were cultured from 10 different, individual female rodents and, we did not manage to isolate *E. coli* from the remaining 54 rodents. *E. coli* was isolated mainly from the gastrointestinal tract (n = 6) and, the trachea and lung (n = 5), with the remaining 2 strains from the liver. A majority of the commensal *E. coli* strains were found in ICR mice (n = 8). Meanwhile, C57BL/6 mice harbored 2 strains and, the BALB/c mouse, Sprague–Dawley and Wistar Kyoto rats, each harbored 1 strain. Two *E*. *coli* strains were isolated from the different organs of 3 individual ICR mice. Strains UM-AEU197 and 198 were isolated from the liver and, the trachea and lung of ICR mouse #1, respectively. Strains UM-AEU202 and 203 were isolated respectively, from the liver and, the trachea and lung of ICR mouse #2. The trachea and lung, and the gastrointestinal tract of ICR mouse #3 sheltered strains UM-AEU213 and 214, respectively (Table 2).

**Table 2:** Genetic characteristics of commensal *E. coli* strains from rodents in the animal experimental unit

Strain	Breed^a^	Organ^b^	Sex^c^	Phylogenetic group	MLST locus								ST
					*dinB*	*icdA*	*pabB*	*polB*	*putP*	*trpA*	*trpB*	*uidA*	
UM-AEU015	SD	GIT	F	A	132	65	3	10	26	53	4	65	746
UM-AEU018	C57BL/6	GIT	F	A	132	65	3	10	26	53	4	65	746
UM-AEU021	C57BL/6	GIT	F	A	5	47	4	10	26	1	4	2	357
UM-AEU116	WKY	GIT	F	A	133	41	47	169	178	23	20	164	747
UM-AEU131	ICR	GIT	F	A	25	65	3	10	180	53	4	65	748
UM-AEU140	BALB/c	T&L	F	A	25	65	48	10	179	8	2	2	749
UM-AEU197	ICR	Liv	F	A	25	65	48	10	179	8	2	2	749
UM-AEU198	ICR	T&L	F	A	25	65	48	10	179	8	2	2	749
UM-AEU202	ICR	Liv	F	A	25	65	48	10	179	8	2	2	749
UM-AEU203	ICR	T&L	F	A	25	65	48	10	179	8	2	2	749
UM-AEU208	ICR	T&L	F	A	25	65	48	10	179	8	2	2	749
UM-AEU213	ICR	T&L	F	A	25	65	48	10	179	8	2	2	749
UM-AEU214	ICR	GIT	F	A	25	65	48	10	179	8	2	2	749

^a^Breed; *SD* Sprague–Dawley rat, *WKY* Wistar Kyoto rat, *BALB/c* BALB/c mouse, *C57BL/6* C57BL/6 mouse, *ICR* ICR mouse

^b^Organ; *GIT* gastrointestinal tract, *T&L* trachea and lungs, *Liv* liver

^c^Sex; *F* female

Analysis of the commensal *E. coli* strains by MLST revealed 13 strains with 4 novel and 1 common STs (Table 2). The E*. coli* strains recovered from ICR mice, were designated into ST748 and ST749. Strains recovered from Sprague–Dawley and Wistar Kyoto rats were designated into ST746 and ST747, respectively. In addition, ST747 was also found in a C57BL/6 mouse. The sole common *E. coli* ST357 was recovered from the gastrointestinal tract of another C57BL/6 mouse. The gastrointestinal tract was found to harbor all 5 different STs (ST357, ST746, ST747, ST748 and ST749) in this study. The liver and, the trachea and lung however, only harbored ST749 and it was also the most frequent ST encountered. Analysis into the phylogroups of the commensal *E. coli* strains by the quadruplex PCR method revealed that all 13 strains belonged to phylogroup A (Table 2). The alignments of all eight *E. coli* MLST loci relative to the respective reference Allele 1 are shown in Additional files 1, 2, 3, 4, 5, 6, 7 and 8.

The multiplex PCRs to detect the most common clinically significant Shiga toxin-producing *E. coli* serotypes showed that all 13 commensal *E. coli* strains in this study did not belong to any of the serotypes (Additional file 9). Similarly, we observed negative ESBL activity in the 13 commensal *E. coli* strains when tested using the ESBL phenotypic confirmatory method (Table 3). Negative ESBL activity however, does not necessarily mean that the *E. coli* strain is negative for ESBL encoding genes, therefore a series of nucleic acid amplifications for the detection of ESBL encoding genes was performed. However, no ESBL encoding gene was amplified from the commensal *E. coli* strains recovered in our study (Additional files 10, 11, 12).

**Table 3:** Prevalence of ESBL producers among *E. coli* Strains from rodents in the animal experimental unit

Strains	Zone diameter (mm)				ESBL producing organism^b^
	CTX^a^	CTX + CA^a^	CAZ^a^	CAZ + CA^a^	
*K. pneumoniae* ATCC 700603	6	28	7	20	+
*E. coli* ATCC 25922	15	15	16	16	−
UM-AEU015	14	14	14	14	−
UM-AEU018	13	13	15	15	−
UM-AEU021	16	16	16	16	−
UM-AEU116	15	16	15	16	−
UM-AEU131	13	13	14	14	−
UM-AEU140	15	15	15	16	−
UM-AEU197	14	15	15	16	−
UM-AEU198	13	14	14	14	−
UM-AEU202	13	13	13	14	−
UM-AEU203	15	16	16	17	−
UM-AEU208	14	14	15	16	−
UM-AEU213	15	15	16	16	−
UM-AEU214	14	15	15	15	−

^a^CTX = cefotaxime, CTX + CA = cefotaxime-clavulanic acid, CAZ = ceftazidime, CAZ + CA = ceftazidime-clavulanic acid

^b^ESBL producing organism; + = ESBL producer, − = non-ESBL producer

Testing of the animal housing environment, feed and drinking water of the laboratory rodents for the presence of *E. coli* showed that only the wash basin sample resulted in positive *E. coli* culture. The cultured *E. coli* strain belonged to phylogroup B1 and no ESBL genes were detected by PCR. *E. coli* was not cultured from the other animal housing environment samples (table top, tap and trolley), rodent feed and drinking water (Table 4).

**Table 4:** Prevalence of *E. coli* in the animal housing environment, rodent feed and drinking water

Sample	Presence of *E. coli* ^a^	Phylogenetic group^b^	Presence of ESBL genes^c^
Table top	−	−	NP
Tap	−	−	NP
Trolley	−	−	NP
Wash basin	+	B1	−
Rodent feed	−	−	NP
Drinking water	−	−	NP

^a^Presence of *E. coli*; + = *E. coli* present, − = *E. coli* not present

^b^Phylogenetic group; B1 = *E. coli* phylogroup B1, − = not detected

^c^Presence of ESBL genes; − = ESBL genes not detected, NP = not performed

## Discussion

In this study, several key factors involved in influencing the distribution of *E. coli* populations in laboratory rodents were controlled. MacConkey agar was used for the culture of *E*. *coli* strains in this study because it has the propensity to culture members of the *Enterobacteriaceae* and cultured *E. coli* colonies can be easily distinguished by its morphology and color (Gordon and Cowling 2003). Consequently, we only sampled one *E. coli* strain from each suspected agar plate representing the most dominant strain despite evidence that hosts can be colonized with more than one *E. coli* strain at the same time (Gordon et al. 2005). Even though *E. coli* is an established colonizer of the mouse gut (Skurnik et al. 2016), translocation to other organs including the liver (Ohsugi et al. 1996) and the lung (Wu et al. 2010) has been reported. Therefore, in our study, we examined the other rodent organs (liver and, the trachea and lung) for the presence of *E. coli*. In agreement with the previous reports (Ohsugi et al. 1996; Wu et al. 2010), we found two *E*. *coli* strains, each, from different organs in three individual ICR mice. The rodent skin was scrubbed with antiseptic prior to surgery and the harvesting of rodent organs in the following sequence; firstly, the trachea and lung, secondly, the liver, and lastly, the gastrointestinal tract. New sets of sterile surgical tools were utilized for the harvesting of different organs. These procedures were performed to prevent *E*. *coli* cross contamination and translocation was evident with the sighting of similar STs among *E*. *coli* strains from different organs of the same individual.

The distribution of *E. coli* populations in the mammalian host depended on the host habitat, diet, gut anatomy and body mass (Gordon and Cowling 2003). In humans, morphological, physiological and dietary differences among individuals of different sex and age influence the distribution of commensal *E. coli* populations (Gordon et al. 2005). It was further suggested that the exposure to novel *E. coli* strains and the health of the animal might contribute to the temporal variability of commensal *E. coli* populations in animals (Grønvold et al. 2010). Shifting of the commensal *E. coli* population in laboratory rodents can alter experimental outcomes by stressing the rodent’s microbiota into a state of imbalance (Ma et al. 2012) and this is not desired in any animal experimental facilities. Previous study has reported a lower likelihood for the transmission of bacterial plasmids carrying virulence genes to animals housed separately or in small groups, in comparison to animals raised in cramped and dense conditions (Koga et al. 2015). The rodents in this study were housed at a barrier facility in open cages maintained at a constant room temperature, relative humidity and light:dark cycle (Loong et al. 2016). Standard operating procedures including, the wearing of personal protective equipment, working on one animal at a time in the biosafety cabinet, changing gloves after handling a single animal and performing surgeries in a dedicated room away from breeding facilities (Loong et al. 2016) were followed as per required under AAALAC accreditation to prevent introduction of other bacteria species into the studied cohort. To prevent possible transmission from the animal housing environment, several sites in the room where the rodents were housed (table top, tap, trolley and wash basin), were selected to test for the presence of *E. coli*. Similarly, the rodent’s feed and drinking water were also tested for the presence of *E. coli*. We however, found that only the sample from the wash basin grew *E. coli* belonging to phylogroup B1 with no detectable ESBL genes. The presence of *E. coli* phylogroup B1 only at the wash basin suggests that the strain favors the water environment, agreeing with the earlier report by Ratajczak et al. (2010) who found phylogroup B1 strains to be specifically adapted and persistent in water.

The rodents were fed with diet comprising of products made of soy and corn, equivalent to a herbivorous diet. Identical animal feed was maintained throughout the study without the change of vendor or food variant, and the non-alteration of diet preserves the condition in the gut favoring the dominant *E. coli* strain (Blyton et al. 2013). Introduction of different diets would disturb the gut ecosystem leading to the colonization of a different *E. coli* strain (Grønvold et al. 2010). Sexually mature healthy rodents (Kempermann et al. 1997; Sengupta 2013) were selected because the dominant commensal *E. coli* strains would have successfully established stable colonization in the gut flora (Tenaillon et al. 2010) and good health ensured no shifting of host physiological condition (Grønvold et al. 2010), possibly displacing dominant *E. coli* strains in unhealthy animals. The isolation of commensal *E. coli* only from females in this study suggests that host gender could influence the distribution of *E. coli* and this was similar to human *E. coli* strains (Gordon et al. 2005), even though the number of rodent strains in this study was relatively small to draw a definite conclusion. In terms of the gut anatomy, *E. coli* is inclined to flourish in hosts (herbivores and omnivores) with intestinal tracts resembling a microbial fermentation chamber such as a caecum (Gordon and Cowling 2003; Gordon et al. 2005) and all rodents possess a caecum (Gordon and Cowling 2003). In addition, the likelihood of isolating *E. coli* increases with the body mass of animals (Gordon et al. 2005). This was not the case in our study however, where smaller mice contributed to >80 % (11/13) of the isolated commensal *E. coli* strains.

Assessment of the *E. coli* phylogroups categorized the strains isolated from laboratory mice and rats into a single phylogroup A. This finding is largely concordant with other published results on free-range poultry (Koga et al. 2015) and domesticated animals (Tenaillon et al. 2010), where the animals were not housed in dense conditions. Phylogroup B1 strains are best adapted in animals, followed by phylogroup A (Tenaillon et al. 2010), suggesting consistency in our results. The population diversity of *E. coli* due to host diet has been reported with phylogroup A strains predominating in carnivores and omnivores (Escobar-Páramo et al. 2006). Rodents by nature are omnivores and although they were fed a herbivorous diet in this study, it did not change the genetic structure of commensal *E. coli* strains, suggesting the effect of nutrients on the selection of strains. Perhaps the threshold concentration of nutrients could not be achieved from a herbivorous diet, hence phylogroups associated to herbivores could not succeed in colonization (Gordon et al. 2005). The probability of discovering phylogroup A strains is also higher in female humans (Gordon et al. 2005), similar to the findings in laboratory rodents in this study. Another possible explanation for this finding could be that sex hormones may modulate host physiological conditions and it has been demonstrated in humans that sex hormones provoke metabolic changes that shape the intestinal microbiota (Koren et al. 2012). The association between the female gender and *E*. *coli* colonization suggests laboratory rodents to be a good model for studying the impact of sex hormones on the colonization of other commensal bacteria species. Gordon and Cowling (2003) also reported from their survey of the distribution of *E. coli* in Australian vertebrates that phylogroup A can be recovered from any vertebrate group, however this phylogroup is also responsible for causing intestinal infections in mammals. The finding of a different phylogroup B1 from the wash basin further supports our suggestion that the strains recovered from the laboratory rodents were true commensals and transmission from the animal housing environment, feed or water did not occur. The absence of ESBL genes in the *E. coli* strain isolated from the wash basin indicated that the rodent housing facility was not tainted by any clinical bacterial strains carrying resistance genes.

The discovery of 4 novel STs (ST746–ST749) on top of one common ST (ST357) from a relatively small number of specimens using MLST, signified that the rodent’s microbiota is more diverse than the human’s (Smati et al. 2015). Our findings revealed a distinct demarcation of STs for laboratory rodents, with ST357, ST748 and ST749 found in mice, and ST747 in rats (Table 2). This could be explained by the genetic variability among different rodent breeds (Wu et al. 2001) that predisposes them to having different physiological responses (Gibney et al. 2010) and, hormone changes are known to affect bacteria colonization in the host (Blyton et al. 2013). Isolation of ST746 from both mouse and rat suggest that this ST could have adapted to the physiology of different rodent breeds. Based on ST similarities in the different *E. coli* strains cultured from the liver and, the trachea and lung specimens denote that the rodent body is frequently exposed to commensal bacteria from the gastrointestinal tract (Wu et al. 2001). It has been widely accepted that *E. coli* phylogroups are non-randomly distributed among mammalian hosts (Gordon et al. 2005; Clermont et al. 2013) and all of these findings imply that phylogroup A strains establish an ecological niche in laboratory rodents.

Serotyping of the isolated commensal *E. coli* strains using multiplex PCR assays revealed that all the rodent strains were negative for the 21 most clinically relevant Shiga toxin-producing *E. coli*. All commensal *E. coli* strains were also negative for ESBL production and genes encoding beta lactamases. This was not surprising, considering that the rodents were housed in proper AAALAC accredited animal housing facility. The level of hygiene in the human population was noted as one of the main factors for the significant shift in the proportions of *E. coli* strains in France (Tenaillon et al. 2010) and an increased level of antimicrobial resistance in *E. coli* isolated from animals was observed with an increase density of human population living in close proximity to these animals (Skurnik et al. 2016). Furthermore, we could not culture *E. coli* from a large number of our specimens, suggesting that the bacteria density was very low in the organs and low bacteria density would restrict the occurrence of horizontal resistance gene transfer (Skurnik et al. 2016). The low bacteria density and the absence of antimicrobial resistance genes in commensal *E. coli* strains of laboratory rodents is consistent with the hypothesis that strains with more virulence genes are likely to command better survival fitness and persist longer in the gut (Blyton et al. 2013). One limitation of this study was the relatively small number of isolated strains and the study was performed in only one animal experimental facility. It is possible that *E. coli* strains from other animal species and other animal experimental facilities would differ.

## Conclusions

This study found that the commensal *E. coli* strains isolated from different rodent breeds housed in the Animal Experimental Unit, Faculty of Medicine, University of Malaya were categorized as members of phylogroup A. Four novel sequence types were uncovered using MLST, displaying the broad diversity of commensal *E. coli* strains from laboratory rodents. As expected, the isolated *E. coli* strains did not possess antibiotic resistance genes and, were not Shiga toxin and extended spectrum beta lactamase producers. We propose that animal experimental facilities should initiate surveillance of commensal *E. coli* strains for the determination of phylogroups and the presence of ESBL genes, to monitor for events that could lead to dysbiosis and distortion of animal experimental data. Taken together, the absence of antimicrobial resistance and virulence genes in the *E. coli* cohort of this study indicate the true commensal nature of these strains. To the best of our knowledge, this study is also the first to analyze the genetic structure of commensal *E. coli* in laboratory rodents.

## Additional files

10.1186/s40064-016-2745-9 Sequence alignments of *E. coli* MLST *dinB* loci. Allelic profiles of commensal *E. coli* were compared against Allele 1 (Ref-dinB) and positions at which differences were found relative to Allele 1, were noted above the reference allele. Each position represents point mutations relative to the reference allele. Nucleotide differences were displayed whereas black dots represent nucleotides similar to the reference allele.
10.1186/s40064-016-2745-9 Sequence alignments of *E. coli* MLST *icdA* loci. Allelic profiles of commensal *E. coli* were compared against Allele 1 (Ref-icdA) and positions at which differences were found relative to Allele 1, were noted above the reference allele. Each position represents point mutations relative to the reference allele. Nucleotide differences were displayed whereas black dots represent nucleotides similar to the reference allele.
10.1186/s40064-016-2745-9 Sequence alignments of *E. coli* MLST *pabB* loci. Allelic profiles of commensal *E. coli* were compared against Allele 1 (Ref-pabB) and positions at which differences were found relative to Allele 1, were noted above the reference allele. Each position represents point mutations relative to the reference allele. Nucleotide differences were displayed whereas black dots represent nucleotides similar to the reference allele.
10.1186/s40064-016-2745-9 Sequence alignments of *E. coli* MLST *polB* loci. Allelic profiles of commensal *E. coli* were compared against Allele 1 (Ref-polB) and positions at which differences were found relative to Allele 1, were noted above the reference allele. Each position represents point mutations relative to the reference allele. Nucleotide differences were displayed whereas black dots represent nucleotides similar to the reference allele.
10.1186/s40064-016-2745-9 Sequence alignments of *E. coli* MLST *putP* loci. Allelic profiles of commensal *E. coli* were compared against Allele 1 (Ref-putP) and positions at which differences were found relative to Allele 1, were noted above the reference allele. Each position represents point mutations relative to the reference allele. Nucleotide differences were displayed whereas black dots represent nucleotides similar to the reference allele. Hyphens represent deletion at that particular allele.
10.1186/s40064-016-2745-9 Sequence alignments of *E. coli* MLST *trpA* loci. Allelic profiles of commensal *E. coli* were compared against Allele 1 (Ref-trpA) and positions at which differences were found relative to Allele 1, were noted above the reference allele. Each position represents point mutations relative to the reference allele. Nucleotide differences were displayed whereas black dots represent nucleotides similar to the reference allele.
10.1186/s40064-016-2745-9 Sequence alignments of *E. coli* MLST *trpB* loci. Allelic profiles of commensal *E. coli* were compared against Allele 1 (Ref-trpB) and positions at which differences were found relative to Allele 1, were noted above the reference allele. Each position represents point mutations relative to the reference allele. Nucleotide differences were displayed whereas black dots represent nucleotides similar to the reference allele.
10.1186/s40064-016-2745-9 Sequence alignments of *E. coli* MLST *uidA* loci. Allelic profiles of commensal *E. coli* were compared against Allele 1 (Ref-uidA) and positions at which differences were found relative to Allele 1, were noted above the reference allele. Each position represents point mutations relative to the reference allele. Nucleotide differences were displayed whereas black dots represent nucleotides similar to the reference allele.
10.1186/s40064-016-2745-9 Gel electrophoresis images Shiga toxin-producing *E. coli* serotypes. A) Multiplex PCR #1. Positive control = O103; O5, O26, O91, O111, O121 and O145 positive controls were not included as we do not have strains encoding those genes. B) Multiplex PCR #2. Positive control = O76; O45, O55, O113, O128, O146 and O177 positive controls were not included as we do not have strains encoding those genes. C) Multiplex PCR #3. Positive control = O118; O15, O104, O123, O157, O165 and O172 positive controls were not included as we do not have strains encoding those genes. - = negative control, 1 = UM-AEU015, 2 = UM-AEU018, 3 = UM-AEU021, 4 = UM-AEU116, 5 = UM-AEU131, 6 = UM-AEU140, 7 = UM-AEU197, 8 = UM-AEU198, 9 = UM-AEU202, 10 = UM-AEU203, 11 = UM-AEU208, 12 = UM-AEU213, 13 = UM-AEU214.
10.1186/s40064-016-2745-9 Gel electrophoresis images of genes encoding for ESBL. A) CTX-M multiplex PCR. Positive control = CTX-M group 9; other CTX-M groups 1, 2, 8 and 25 positive controls were not included as we do not have strains encoding those genes. B) TEM/SHV/OXA-1-like multiplex PCR. Positive control = TEM and SHV; OXA-1-like positive control was not included as we do not have strains encoding that gene. - = negative control, 1 = UM-AEU015, 2 = UM-AEU018, 3 = UM-AEU021, 4 = UM-AEU116, 5 = UM-AEU131, 6 = UM-AEU140, 7 = UM-AEU197, 8 = UM-AEU198, 9 = UM-AEU202, 10 = UM-AEU203, 11 = UM-AEU208, 12 = UM-AEU213, 13 = UM-AEU214.
10.1186/s40064-016-2745-9 Gel electrophoresis images of genes encoding for ESBL. A) Plasmid-mediated AmpC gene multiplex PCR. Positive control = LAT-1; ACC, FOX, MOX, DHA and EBC positive controls were not included as we do not have strains encoding those genes. B) VEB/GES/PER multiplex PCR. Positive control = GES; VEB and PER positive controls were not included as we do not have strains encoding those genes. - = negative control, 1 = UM-AEU015, 2 = UM-AEU018, 3 = UM-AEU021, 4 = UM-AEU116, 5 = UM-AEU131, 6 = UM-AEU140, 7 = UM-AEU197, 8 = UM-AEU198, 9 = UM-AEU202, 10 = UM-AEU203, 11 = UM-AEU208, 12 = UM-AEU213, 13 = UM-AEU214.
10.1186/s40064-016-2745-9 Gel electrophoresis images of genes encoding for ESBL. A) VIM/IMP/KPC multiplex PCR. Positive control = IMP; VIM and KPC positive controls were not included as we do not have strains encoding those genes. B) OXA-48-like PCR. Positive control = OXA-48. - = negative control, 1 = UM-AEU015, 2 = UM-AEU018, 3 = UM-AEU021, 4 = UM-AEU116, 5 = UM-AEU131, 6 = UM-AEU140, 7 = UM-AEU197, 8 = UM-AEU198, 9 = UM-AEU202, 10 = UM-AEU203, 11 = UM-AEU208, 12 = UM-AEU213, 13 = UM-AEU214. Since we do not have positive control for NDM-1, the gel electrophoresis image was not presented.
