# Supplementary material for: Genetic characterization of commensal Escherichia coli isolated from laboratory rodents
Source: Springerplus. 2016 Jul 11;5(1):1035. doi: 10.1186/s40064-016-2745-9 (PMC4940358; doi:10.1186/s40064-016-2745-9)
Supplement: Supplementary file 6 — 10.1186/s40064-016-2745-9 Sequence alignments of E. coli MLST trpA loci. Allelic profiles of commensal E. coli were compared against Allele 1 (Ref-trpA) and positions at which differences were found relative to Allele 1, were noted above the reference allele. Each position represents point mutations relative to the reference allele. Nucleotide differences were displayed whereas black dots represent nucleotides similar to the reference allele. [file 40064_2016_2745_MOESM6_ESM.docx]

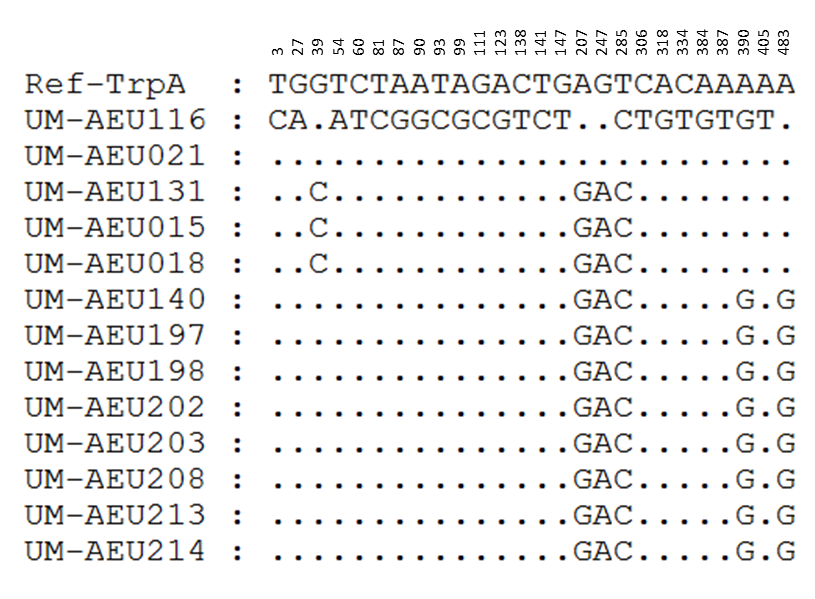


Additional file 6: Sequence alignments of *E. coli* MLST *trpA* loci. Allelic profiles of commensal *E. coli* were compared against Allele 1 (Ref-trpA) and positions at which differences were found relative to Allele 1, were noted above the reference allele. Each position represents point mutations relative to the reference allele. Nucleotide differences were displayed whereas black dots represent nucleotides similar to the reference allele.
