# Supplementary material for: Genetic characterization of commensal Escherichia coli isolated from laboratory rodents
Source: Springerplus. 2016 Jul 11;5(1):1035. doi: 10.1186/s40064-016-2745-9 (PMC4940358; doi:10.1186/s40064-016-2745-9)
Supplement: Supplementary file 9 — 10.1186/s40064-016-2745-9 Gel electrophoresis images Shiga toxin-producing E. coli serotypes. A) Multiplex PCR #1. Positive control = O103; O5, O26, O91, O111, O121 and O145 positive controls were not included as we do not have strains encoding those genes. B) Multiplex PCR #2. Positive control = O76; O45, O55, O113, O128, O146 and O177 positive controls were not included as we do not have strains encoding those genes. C) Multiplex PCR #3. Positive control = O118; O15, O104, O123, O157, O165 and O172 positive controls were not included as we do not have strains encoding those genes. - = negative control, 1 = UM-AEU015, 2 = UM-AEU018, 3 = UM-AEU021, 4 = UM-AEU116, 5 = UM-AEU131, 6 = UM-AEU140, 7 = UM-AEU197, 8 = UM-AEU198, 9 = UM-AEU202, 10 = UM-AEU203, 11 = UM-AEU208, 12 = UM-AEU213, 13 = UM-AEU214. [file 40064_2016_2745_MOESM9_ESM.docx]

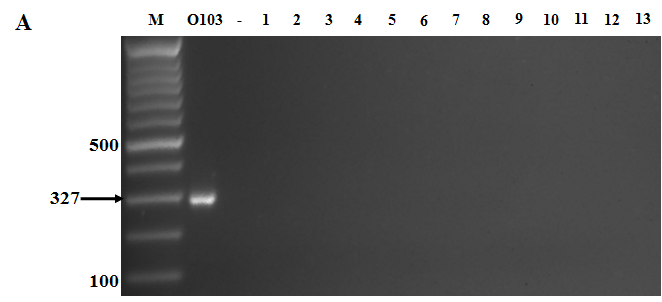


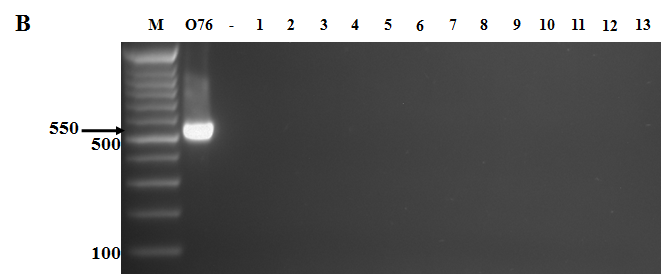


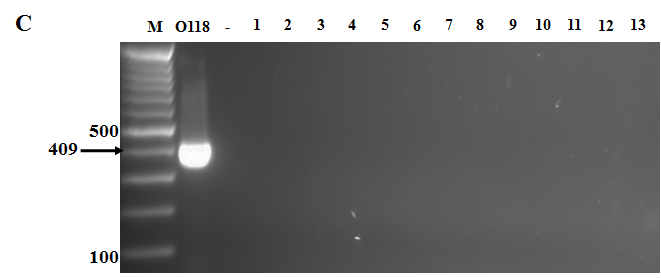


Additional file 9: Gel electrophoresis images Shiga toxin-producing *E. coli* serotypes. A) Multiplex PCR #1. Positive control = O103; O5, O26, O91, O111, O121 and O145 positive controls were not included as we do not have strains encoding those genes. B) Multiplex PCR #2. Positive control = O76; O45, O55, O113, O128, O146 and O177 positive controls were not included as we do not have strains encoding those genes. C) Multiplex PCR #3. Positive control = O118; O15, O104, O123, O157, O165 and O172 positive controls were not included as we do not have strains encoding those genes. - = negative control, 1 = UM-AEU015, 2 = UM-AEU018, 3 = UM-AEU021, 4 = UM-AEU116, 5 = UM-AEU131, 6 = UM-AEU140, 7 = UM-AEU197, 8 = UM-AEU198, 9 = UM-AEU202, 10 = UM-AEU203, 11 = UM-AEU208, 12 = UM-AEU213, 13 = UM-AEU214.
