# Supplementary material for: Genetic characterization of commensal Escherichia coli isolated from laboratory rodents
Source: Springerplus. 2016 Jul 11;5(1):1035. doi: 10.1186/s40064-016-2745-9 (PMC4940358; doi:10.1186/s40064-016-2745-9)
Supplement: Supplementary file 11 — 10.1186/s40064-016-2745-9 Gel electrophoresis images of genes encoding for ESBL. A) Plasmid-mediated AmpC gene multiplex PCR. Positive control = LAT-1; ACC, FOX, MOX, DHA and EBC positive controls were not included as we do not have strains encoding those genes. B) VEB/GES/PER multiplex PCR. Positive control = GES; VEB and PER positive controls were not included as we do not have strains encoding those genes. - = negative control, 1 = UM-AEU015, 2 = UM-AEU018, 3 = UM-AEU021, 4 = UM-AEU116, 5 = UM-AEU131, 6 = UM-AEU140, 7 = UM-AEU197, 8 = UM-AEU198, 9 = UM-AEU202, 10 = UM-AEU203, 11 = UM-AEU208, 12 = UM-AEU213, 13 = UM-AEU214. [file 40064_2016_2745_MOESM11_ESM.docx]

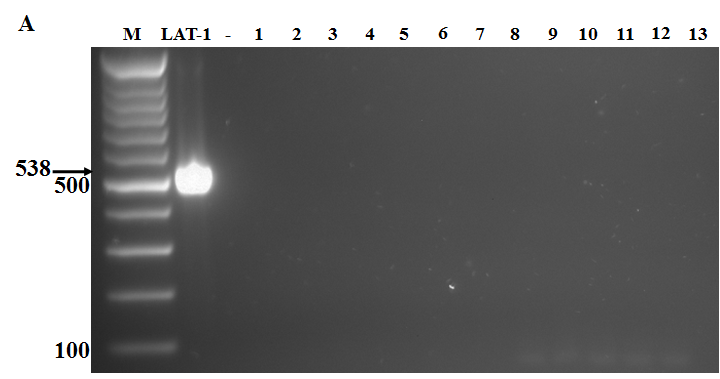


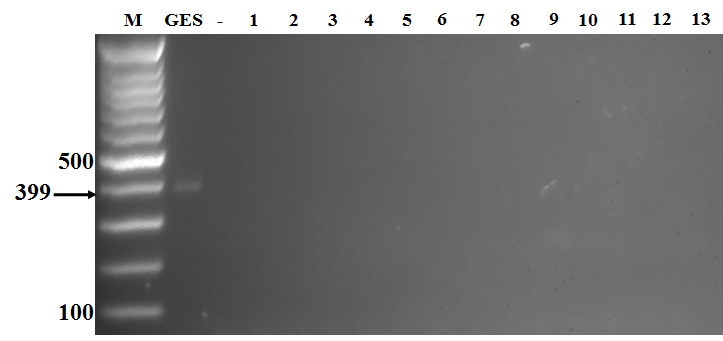


Additional file 11: Gel electrophoresis images of genes encoding for ESBL. A) Plasmid-mediated AmpC gene multiplex PCR. Positive control = LAT-1; ACC, FOX, MOX, DHA and EBC positive controls were not included as we do not have strains encoding those genes. B) VEB/GES/PER multiplex PCR. Positive control = GES; VEB and PER positive controls were not included as we do not have strains encoding those genes. - = negative control, 1 = UM-AEU015, 2 = UM-AEU018, 3 = UM-AEU021, 4 = UM-AEU116, 5 = UM-AEU131, 6 = UM-AEU140, 7 = UM-AEU197, 8 = UM-AEU198, 9 = UM-AEU202, 10 = UM-AEU203, 11 = UM-AEU208, 12 = UM-AEU213, 13 = UM-AEU214.
